# Characterization of Thymic Nurse-Cell Lymphocytes, Using an Improved Procedure for Nurse-Cell Isolation

**DOI:** 10.1155/1993/43482

**Published:** 1993

**Authors:** Mireille Lahoud, David Vremec, Richard L. Boyd, Ken Shortman

**Affiliations:** 1 The Walter and Eliza Hall Institute of Medical Research Melbourne Victoria 3050 Australia; 2 Department of Pathology and Immunology Monash University Medical School prahan Victoria 3181 Australia

**Keywords:** Thymic nurse cells, multicellular complexes, thymus microenvironments, T-cell maturation, positive selection, cell separation, cell elutriation

## Abstract

Thymic nurse cells (TNC), multicellular complexes consisting of lymphoid cells enclosed within cortical epithelial cells, were isolated from mouse thymus by a modified procedure allowing immunofluorescent labeling and flow cytometric analysis of their lymphoid contents (TNC-L). Collagenase was the only protease used for tissue digestion, to ensure that surface antigen markers remained intact. Zonal unit-gravity elutriation was used to enrich the TNC on the basis of their high sedimentation rate, followed by immunomagnetic bead depletion to remove residual mononuclear cell contaminants and a density separation to remove debris. The TNC-L were then released from inside TNC by a short period of culture. The measured contamination of TNC-L with exogenous thymocytes was around 0.5%. Three-color immunofluorescent labeling revealed that TNC-L included, as well as a maiority of immature CD4^+^8^+^3^low^ thymocytes, about 12% of apparently mature CD4^+^8^-^3^high^ and CD4^-^8^+^3^high^ thymocytes. TNC are located in the cortex, where mature cells are rare; the occurrence of mature phenotype cells within these structures suggests that they represent a microenvironment for the selection and generation of mature T cells.


*Developmental Immunology*, 1993, Vol. 3, pp. 103–112
Reprints available directly from the publisher
Photocopying permitted by license only

© 1993 Harwood Academic Publishers GmbH
Printed in the United States of America

### Characterization of Thymic Nurse-Cell Lymphocytes, Using an Improved Procedure for Nurse-Cell Isolation

MIREILLE LAHOUD,† DAVID VREMEC,† RICHARD L. BOYD,‡ and KEN SHORTMAN*†

†*The Walter and Eliza Hall Institute of Medical Research, Melbourne, Victoria 3050, Australia*
‡*Department of Pathology and Immunology, Monash University Medical School, Prahan, Victoria 3181, Australia*

Thymic nurse cells (TNC), multicellular complexes consisting of lymphoid cells enclosed within cortical epithelial cells, were isolated from mouse thymus by a modified procedure allowing immunofluorescent labeling and flow cytometric analysis of their lymphoid contents (TNC-L). Collagenase was the only protease used for tissue digestion, to ensure that surface antigen markers remained intact. Zonal unit-gravity elutriation was used to enrich the TNC on the basis of their high sedimentation rate, followed by immunomagnetic bead depletion to remove residual mononuclear cell contaminants and a density separation to remove debris. The TNC-L were then released from inside TNC by a short period of culture. The measured contamination of TNC-L with exogenous thymocytes was around 0.5%. Three-color immunofluorescent labeling revealed that TNC-L included, as well as a majority of immature $CD4^{+}8^{+}3^{low}$ thymocytes, about 12% of apparently mature $CD4^{+}8^{-}3^{high}$ and $CD4^{-}8^{+}3^{high}$ thymocytes. TNC are located in the cortex, where mature cells are rare; the occurrence of mature phenotype cells within these structures suggests that they represent a microenvironment for the selection and generation of mature T cells.

KEYWORDS: Thymic nurse cells, multicellular complexes, thymus microenvironments, T-cell maturation, positive selection, cell separation, cell elutriation.

### INTRODUCTION

Thymic nurse cells (TNC), multicellular complexes isolated from the thymus following enzymic digestion, consist of 10–200 thymocytes enclosed within a cortical epithelial cell (reviewed by Kyewski, 1986). Since their first description by Wekerle and Ketelson (1980; Wekerle et al., 1980), the intimate interaction implied by this structure has prompted suggestions that the epithelial component is inducing early steps in T-cell differentiation or is mediating positive or negative selection to shape the T-cell antigen-receptor (TCR) repertoire. Much depends on whether the TNC represents a prexistent secluded microenvironment sequestering a distinct subset of cortical thymocytes or whether the TNC merely represents a postdigestion folding structure that has captured a random sample of cortical thymocytes. There is good evidence that the association between the epithelial cell and the thymocytes precedes the digestion step, although the issue of whether this is in the form of an open or a closed structure *in situ* is not settled. The nature of the thymocytes within the nurse cell has also been a subject of dispute.

There is general agreement that the majority of nurse-cell associated lymphocytes (TNC-L) are of the $CD4^{+}8^{+}$ type, as are most cortical thymocytes (reviewed by Kyewski, 1986). Further clues to nurse-cell function, therefore, depend on our ability to analyze the minority TNC-L components, and determine whether early precursor cells or late, mature thymocytes are present. Our earlier studies on single micromanipulated murine TNC (Andrews et al., 1985) indicated that about 3% of TNC-L showed a proliferative clonal response to mitogens, but that none of these clones was cytotoxic; this suggested the presence of mature $CD4^{+}8^{-}$ thymocytes, but not of mature $CD4^{-}8^{+}$ thymocytes. In contrast, functional allo-specific cytotoxic precursors have been detected by Fink et al. (1984) in bulk preparations of TNC-

*Corresponding author.

L. Recently, Penninger et al. (1990), using direct two-color immunofluorescent staining of lymphocytes within chicken nurse cells, found a high incidence of both CD4$^{+}$8$^{-}$ and CD4$^{-}$8$^{+}$ thymocytes, suggesting mature cells of both cytotoxic and helper lineages are present. However, in view of the relatively high incidence of "immature single positives" in the chicken thymus (Davidson and Boyd, 1992), a third fluorescent color to stain the CD3-TDR complex would have been required unequivocally to identify mature cells.

There have been several obstacles to the direct flow cytometric analysis of the thymocytes released from TNC. Rigorous exclusion of exogenous thymocyte contaminants is required before minor subsets within the already small TNC-L yield can be detected with confidence. The use of proteases such as trypsin in the digestion procedure cleaves surface markers from thymocytes (even from those within TNC), preventing useful immunofluorescent labeling. The use of collagenase alone in the digestion procedure can avoid the loss of surface antigens, but such digests usually include fibrous material that interferes with TNC purification and gives a poor TNC yield; the resultant TNC then have proportionally higher contaminant levels. We now present a modified procedure for isolating murine TNC and TNC-L with surface markers intact, in yield and purity adequate for flow cytometric analysis. We now find apparently mature cells of both the CD4$^{-}$8$^{+}$ and CD4$^{+}$8$^{-}$ lineages within murine TNC.

### RESULTS

### Thymus Disruption and Digestion

Our final procedure for releasing nurse cells from the thymus (Materials and Methods) was based on earlier protocols (Kyewski and Kaplan, 1982; Boyd et al., 1984; Andrews and Boyd, 1985), but with several modifications. Mechanical disruption and extensive washing were used to release as many free thymocytes as possible, to avoid overloading the subsequent elutriation step: About 75% of the free thymocytes were removed at this stage. To avoid cleaving surface antigenic markers from thymocytes, the remaining thymic stroma was digested with collagenase as the only protease (together with DNAse to avoid aggregation); several commercial collagenase preparations were tested, and only those leaving the trypsin-sensitive CD4 and CD8 markers intact after 30 min at 33°C incubation were considered suitable (Fig. 1). After digestion of the thymic stroma with collagenase-DNAse (~20 min, 32°C), a brief incubation with the chelating agent EDTA was used to disrupt thymic rosettes (Kyewski et al., 1987; Shortman et al., 1989), which otherwise separate with some of the smaller-sized nurse cells.

### Elutriation Separation of Nurse Cells

When attempts were made to recover cells from the thymic stromal digest by centrifugation, the

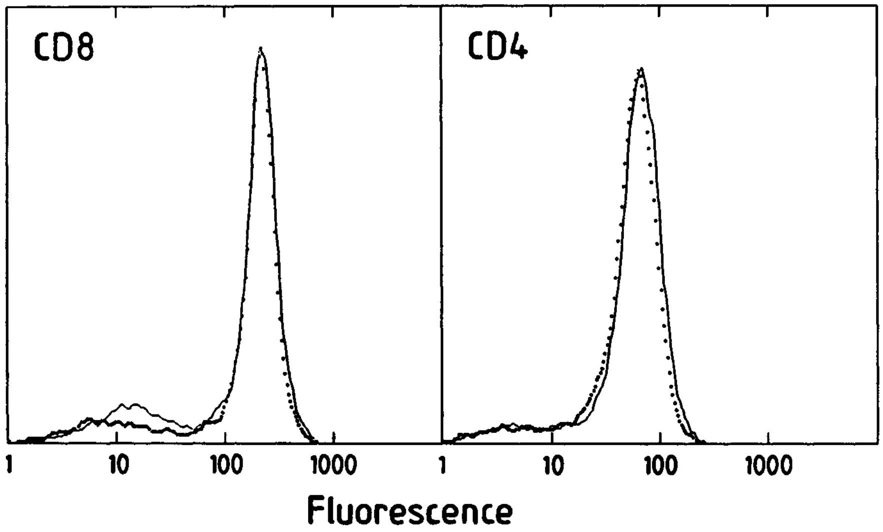

FIGURE 1. The maintenance of surface CD4 and CD8 after collagenase treatment. A suspension of thymocytes was incubated, at 33°C for 25 min, in the collagenase–DNAase digestion mixture to mimic the conditions of TNC isolation. The thymocytes were then washed and stained with mAb-recognizing CD4 or CD8. The vertical axis gives the relative number of cells (arbitrary units, linear scale), and the horizontal axis the relative fluorescence intensity (arbitrary units, logarithmic scale). The dotted line gives the fluorescence staining of thymocytes before incubation, and the continuous line the staining after collagenase treatment.

resulting cell pellet contained fibrous material and aggregates of cells that interfered with subsequent purification. Accordingly, the digest was elutriated directly, without prior cell concentration or washing, so as to produce almost complete purification in a single step. The unit-gravity zonal elutriation procedure described previously (Andrews and Shortman, 1985; Shortman et al., 1989) was used, with the modified chamber shape and dimensions given in Fig. 2. The stabilizing gradient for the elutriation medium required modification to cope with the dense and viscous digest introduced as the initial upper cell band. If the number of thymuses processed was below 15, sufficient density stabilization to avoid streaming could just be achieved by gradients of 60–100% serum. Better density stabilization was achieved using gradients of metrizamide of density 1.015 to 1.028 g/cm$^3$. However, the use of low-viscosity metrizamide media allowed cells that entered the elutriating gradient to sediment more rapidly than cells still in the viscous digest starting band, and this reduced the effectiveness of the separation. The final optimized elutriation medium used 50% serum throughout to maintain a roughly constant viscosity, and used varying concentrations of metrizamide to produce the shallow stabilizing density gradient.

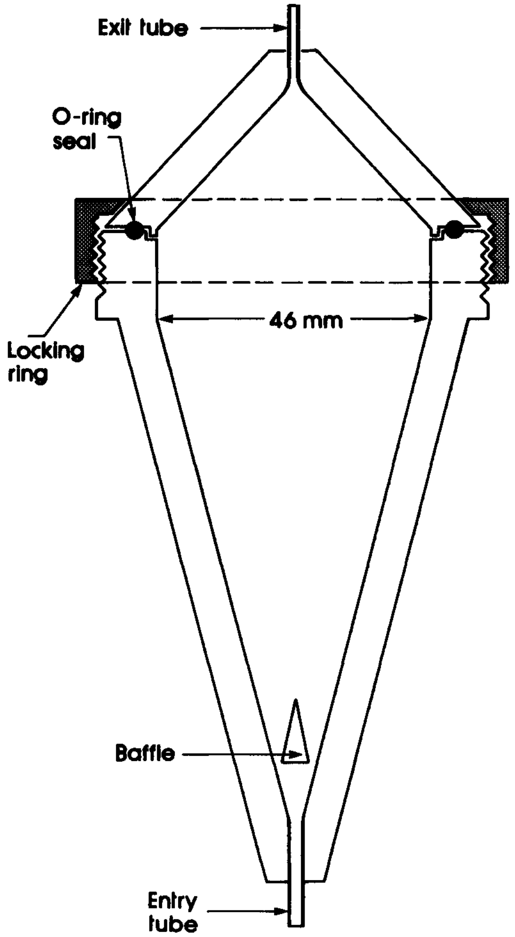

FIGURE 2. Elutriation chamber. The chamber separates into two parts, a lower conical cone and an upper conical exit lid. They are held together by an aluminium locking ring with a screw thread and an O-ring to ensure a tight seal. The transparent chamber is turned from acrylic or cast in epoxy resin. A flat-bottomed cone-shaped baffle is fixed 10 mm above the entry port to dissipate the energy of the entering fluid and prevent disturbance of the medium gradient higher up in the conical chamber. This baffle (11 mm high, 6 mm diameter at the base) is fixed in place by three stainless-steel wire supports; precise centering and placement of the baffle are crucial. Entry and exit tubing is of 2 mm internal diameter stainless-steel tube, flanged to make a smooth exit or entry port. The chamber sits on an aluminium stand, similar to a retort stand, with a 50-mm internal-diameter ring to secure the chamber by the external ridge near the top of the lower cone. Further details of the elutriation system are in Andrews and Shortman (1985).

The wide range of nurse-cell sizes (Fig. 3) led to a wide spread in sedimentation velocities and so a broad elutriation distribution. Nevertheless, a very marked separation from free mononuclear cells was obtained (Fig. 4). In practice, the first TNC-containing fraction overlapped with some large mononuclear cells and was discarded; fractions corresponding to 20–80 ml elutriation volume were pooled. These contained about 80% of the TNC and less than 0.4% of the thymocytes in the elutriate. Some TNC remained in the chamber, but were rejected because of contamination with free thymocytes, clumps, and debris.

Despite the high enrichment, the TNC in the pooled fractions were still not pure. Residual mononuclear cells were treated with a cocktail of mAb specific for thymocytes and myeloid cells, and then removed with anti-Ig-coated magnetic beads; this was carried out on a miniature scale to avoid the use of excessive beads and loss of TNC. As a final step, dead cells, cellular and fibrous debris, and residual magnetic beads, were removed by centrifugation in a dense metrizamide medium. Attempts were made to purify TNC on the basis of buoyant density, but the spread of densities was too wide (1.065–1.086 g/cm$^3$ at 4°C, mouse osmolarity) for any useful separation beyond this simple removal of debris.

### Yield and Purity of Nurse Cells

Final yields of intact nurse cells in five experiments using the complete procedure ranged from 100–1000 per thymus, values about ten-fold lower than those obtained when trypsin was included along with collagenase in the digestion procedure. The final preparations contained from 0.1 to 3 (usually about 1) free thymocytes per TNC. However, some of the TNC obtained were fragile and spontaneously releasing their lymphoid content, so a proportion of the free thymocytes present was nurse-cell derived rather than being contaminants. To determine the true level of contamination with exogenous thymocytes, a separate mechanically dissociated suspension of thymocytes was fluoresceinated by treatment with fluorescein isothiocyanate, and then added to the original collagenase digest to a level of one fluoresceinated thymocyte for each free unlabeled thymocyte present. TNC were then isolated by the standard procedure of elutriation, immunobead depletion, and density separation. The ratio of fluorescent to nonfluorescent thymocytes in the final TNC preparation (determined by fluroescence microscopy) averaged 1:5.1, as opposed to the starting ratio of 1:1.0. Thus, exogenous thymocyte contaminants had been diluted with nonfluorescent thymocytes released from TNC. By correcting for this effect, the true level of exogenous thymocyte contaminants ranged from 0.01 to 0.5 thymocytes per TNC.

### Release of Lymphocytes from Nurse Cells

Numerous enzymic, immunological, and mechanical procedures were tested for release of TNC-L from TNC. Most were unsuccessful. In our hands, these failures included the use of anti-Ia mAb and complement (Kyewski, 1986), which gave only partial release and destroyed a proportion of TNC-L that apparently expressed or picked up surface IA. Accordingly, we allowed TNC to release their contents spontaneously, by culturing in a medium optimized for support of

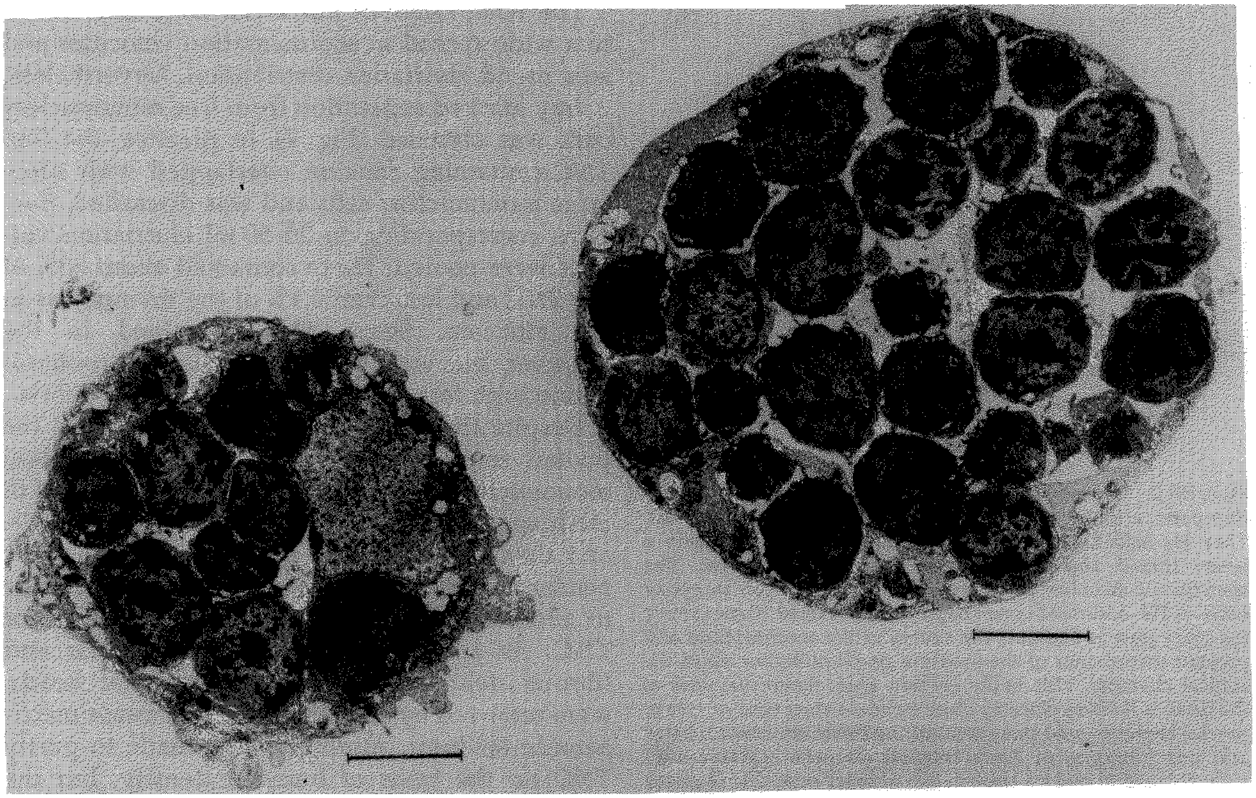

FIGURE 3. A small and a large thymic nurse cell, examples of TNC found in different elutriation fractions. The scale marker represents 5 $\mu$. The epithelial nucleus can be seen in the section of the smaller TNC. The larger TNC may not be completely closed; some such partially open TNC discharge their thymocyte contents during isolation.

T-cell viability. The rate at which nurse cells released their contents varied from nurse cell to nurse cell and from one preparation to another, but was complete in 5–7 hr (Fig. 5). Incubation beyond 7 hr was avoided because of thymocyte death, and all analyses presented were from 7-hr harvests. At this point, most TNC-L recovered were viable (87%). The yield of TNC-L averaged 35 viable thymocytes per nurse cell cultured. The expected level of contamination of these with exogenous thymocytes was, from the previous results, at most 1.5% and generally around 0.5%.

### Surface Phenotype of Nurse-Cell Lymphocytes

The yield of TNC-L from 10–20 thymuses was adequate for immunofluorescent staining and flow cytometric analysis. Because the 7-hr incubation may have affected the surface-marker distribution, the staining of TNC-L was compared to

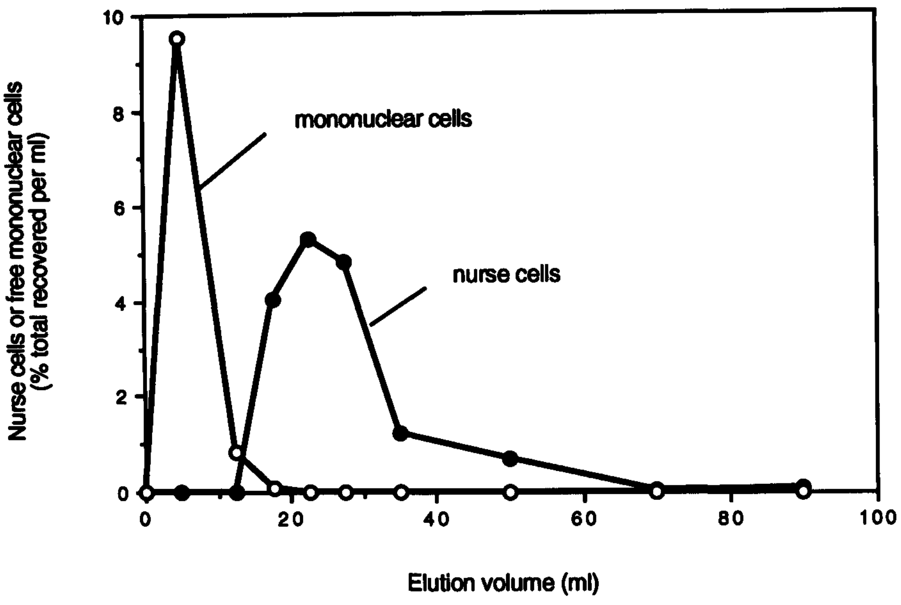

FIGURE 4. The separation of thymic nurse cells from thymocytes and other free mononuclear cells by zonal unit-gravity elutriation. The collagenase digest of thymic stroma (6 ml) was placed in the chamber illustrated in Fig. 2, and elutriated by pumping in medium (stabilized by a shallow density gradient) at a rate of 180 ml/hr. Fractions were collected from out the top of the chamber. Results are typical of four such separations analyzed in detail, before a simple pool of nurse-cell-enriched fractions was adopted.

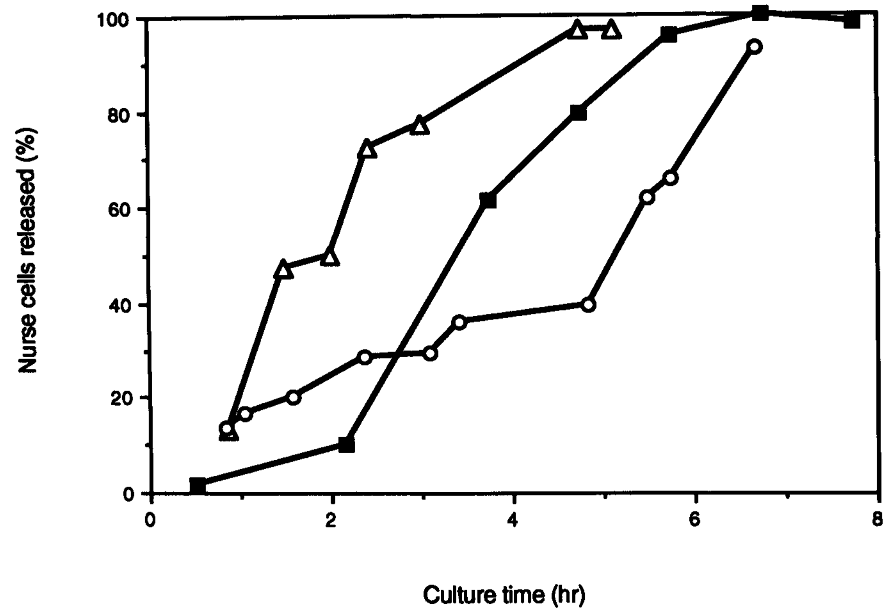

FIGURE 5. The rate of release of thymocytes from nurse cells in culture. Purified TNC were incubated at 37°C in a simple culture medium, based on RPMI-1640, and the number of nurse cells that had discharged their contents counted at various times by inverted phase-contrast microscopy. Three separate experiments are shown to illustrate the variation in release rate.

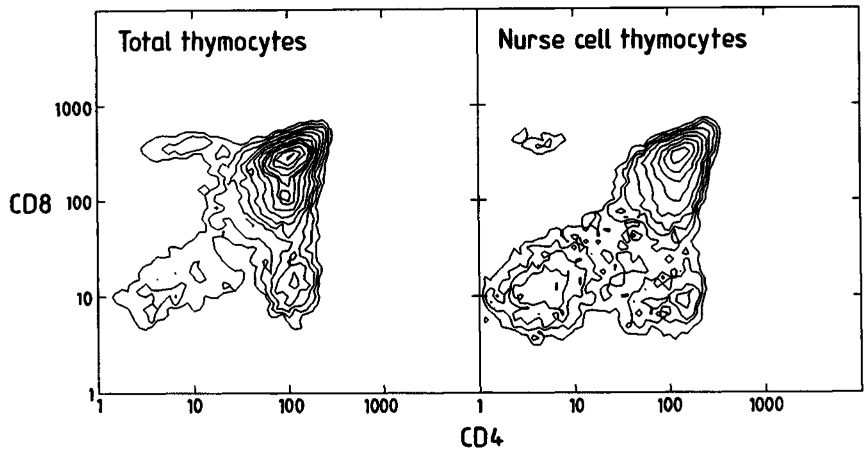

FIGURE 6. The surface antigenic phenotype of thymocytes released from nurse cells (TNC-L). TNC were isolated by collagenase digestion, elutriation, and immunomagnetic bead depletion as in Materials and Methods, and then their lymphoid content collected by incubation for 7 hr as in Fig. 5. The released TNC-L were stained with directly conjugated mAb against CD4 and CD8, and the fluorescence distribution determined. The TNC-L were compared to a suspension of free thymocytes, obtained from the same thymuses, incubated for the same time in the same medium used for TNC-L release. Results are from a single preparation made from a pool of 16 thymuses. Three such experiments gave similar results.

a free thymocyte suspension incubated side by side for the same period. Analysis for CD4 and CD8 expression revealed that all four subpopulations defined by these parameters were present in TNC-L (Figs. 6 and 7). As expected, the dominant population was $CD4^+8^+$ (a mean of 63% in TNC-L compared to 77% in incubated thymocytes). $CD4^-8^-$ cells were also present (a mean of 18% in TNC-L compared to 7% in incubated thymocytes); the analysis was side scatter gated to exclude very large cells, but no other markers have been used to ensure these "double negatives" were T-lineage cells. TNC-L also included single positives, both $CD4^+8^-$ (a mean of 8.7% compared to 7.7% in incubated thymocytes) and $CD4^-8^+$ (a mean of 2.9% compared to 2.7% in incubated thymocytes).

Single positive thymocytes may include, as well as mature cells, earlier immature blast cells of both the $CD4^+8^-$ and $CD4^-8^+$ type (reviewed by Hugo and Petrie, 1991). However, the low-angle light-scatter characteristics of the single positives amongst TNC-L were those of mature thymocytes rather than of early blast cells (data not shown). To check the maturity of these cells more critically, TNC-L were stained for CD3 expression, as well as for CD4 and CD8 (Fig. 7). The distribution of CD3 on TNC-L was similar to that of thymocytes overall, and included cells with no detectable CD3, cells with the low levels characteristic of $CD4^+8^+$ thymocytes, and a smaller proportion of cells with high, mature T-cell levels. When these $CD3^{high}$ cells from TNC were gated and analyzed for CD4 and CD8 expression (Fig. 7), the existence of both the $CD4^-8^+3^{++}$ and the $CD4^+8^-3^{++}$ populations was evident, as well as some intermediates between these mature cells and the $CD4^+8^+$ population.

### DISCUSSION

The isolation procedure we have developed, although giving a relatively low yield of nurse cells because of the use of collagenase as the only proteolytic enzyme, allowed us to analyze the lymphoid contents of nurse cells using a multiparameter immunofluorescent flow cytometric approach. Protease-sensitive surface markers such as CD4 and CD8 remained intact, and the level of exogenous thymocyte contaminants was sufficiently low that even minority components of the TNC-L could be analyzed with confidence.

All four thymic populations defined by CD4 and CD8 appear to be present in TNC. Nurse cells are located in the outer cortex (Kyewski and Kaplan, 1982; van Vliet et al., 1984; Andrews and Boyd, 1985; Kyewski, 1986), and the predominance of $CD4^+8^+$ cells is expected in this location. The predominance of $CD4^+8^+$ cells was also expected from earlier studies (Kyewski and Kaplan, 1982; van Vliet et al., 1984; Andrews et al., 1985; Penninger et al., 1990). The presence of a significant level of $CD4^-8^-$ cells is also in accordance with an outer cortical population. This would fit with nurse cells promoting early

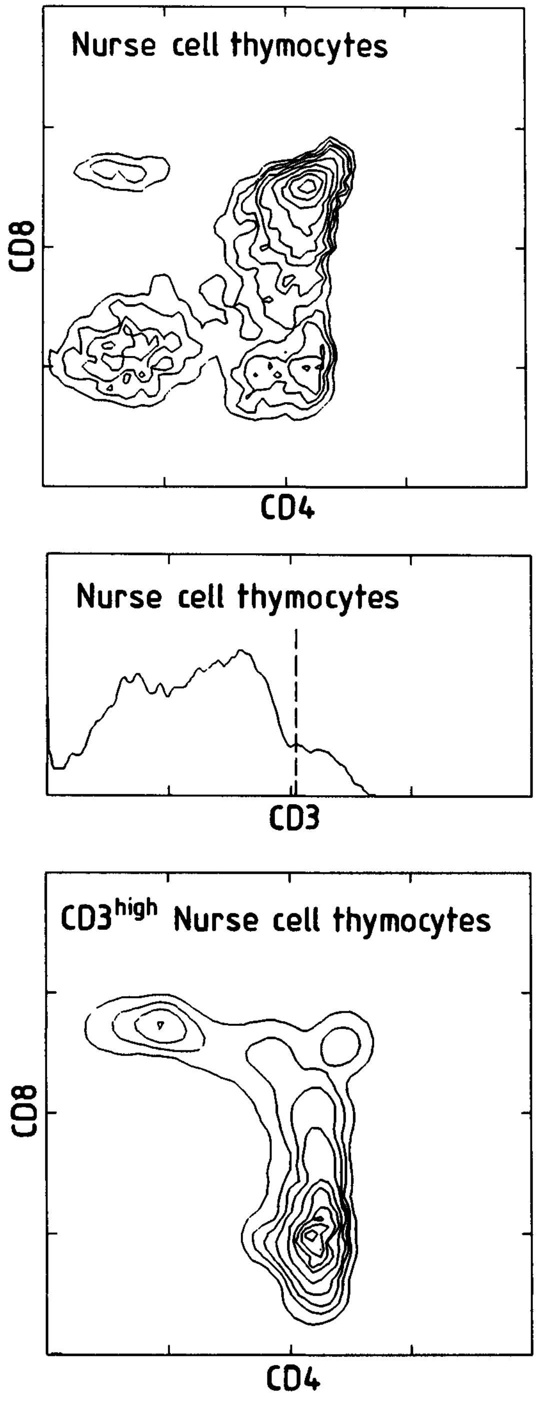

FIGURE 7. The expression of CD3 by lymphocytes released from nurse cells. The experiment was similar to Fig. 6, except three-color immunofluorescent staining was used. The lower CD4×CD8 fluorescence distribution profile was gated, as shown in the centre panel, to show only the phenotype of cells expressing high levels of CD3. All fluorescence distributions are on a four-decade logarithmic scale. Two such experiments gave similar results.

steps in T-cell development, but not with an expectation that their role is limited to the process of repertoire selection (Kyewski, 1986). However, other markers are needed to establish precisely which T-cell developmental stages are present in TNC, and whether these $CD4^-8^-$ cells include cells of the $\gamma\delta$TCR lineage. Without further studies, it is not even certain that the $CD4^-8^-$ cells we obtained were T-lineage cells.

The main point established by the present study is the presence in TNC of both $CD4^-8^+3^{++}$ and $CD4^+8^-3^{++}$ thymocytes, which should correspond to mature cells of both the class I and class II MHC-restricted lineages. This agrees with results obtained with chicken TNC (Penninger et al., 1990), although in this study, the limitation to two fluorescent colors for *in situ* staining did not allow full identification of mature single positive cells. It should be remembered that our results are obtained after a 7-hr incubation to release TNC-L, so some maturation may have occurred during this period; however, TNC must at least have included the immediate postselection precursors of these mature cells. Our present results appear to resolve the earlier controversy on whether murine TNC include precursors of cytotoxic T cells as well as precursors of helper T cells (see Introduction). Our present surface-phenotype data indicate cytotoxic precursor cells should be present in TNC, as Fink et al. (1984) suggested. One possible resolution of the discrepancy in the earlier functional data is that the $CD4^-8^+3^{++}$ cells in TNC are not yet fully mature functionally, so they were unable to respond in our mitogen-stimulated single-cell cultures (Andrews et al., 1985), but were able to complete maturation then respond in the particular mix of factors found in the alloantigen-stimulated cultures of Fink et al. (1984).

This presence within outer cortical TNC of thymocytes with the mature surface-phenotype characteristic of the medulla is striking. Transcapsular labeling studies indicate the incidence of mature thymocytes in the outer cortex as a whole is at least fivefold lower than this level (Shortman et al., 1989). Assuming this sample of the cortex is representative of the environment of the nurse cells, the presence of mature thymocytes within TNC argues against a random entrapment of any cells in the local environment. Rather, the results argue for the view that the nurse cell is a specialized site for the generation of mature thymocytes, which then migrate to the medulla. Studies using TCR-transgenic mice, where the incidence of either class I restricted or class II restricted mature thymocytes can readily be manipulated, would help check this model.

### MATERIALS AND METHODS

### Animals

Female CBA/Wehi mice were raised under specific pathogen-free conditions at the Walter and Eliza Hall Institute animal breeding facility. They were used at 4–5 weeks of age, after being left undisturbed for at least 1 week following transport or cage sorting.

### Purification of TNC

The final procedure adopted was as follows. To digest the tissue, thymuses (10–20) were first chopped with fine, sharp scissors in a medium consisting of RPMI-1640 (mouse osmolarity, 308 m. osmolar, and with additional pH 7.2 HEPES buffer) 1% FCS (fetal calf serum, Commonwealth Serum Laboratories, Melbourne) and 0.001% DNAse (DNAse I, grade II, Boehringer Mannheim GmbH, Mannheim). Free thymocytes were washed out from the thymic fragments at 4°C, by repeated pipetting through a wide-bore Pasteur pipette. The fragments were settled (settling could be speeded by accelerating a swing-out centrifuge to 1000 rpm, followed by immediate deceleration), and the supernatant containing released thymocytes removed. This washing procedure was repeated until most of the readily released thymocytes were eliminated. The released thymocyte samples were pooled, washed, and used as the control for later comparison with TNC-L. The washed stromal fragments were then suspended in 5 ml of a freshly made digestion medium consisting of RPMI-1640 containing 1% FCS, 0.01% DNAse, and 5 mg/ml of selected collagenase (type II; Worthington Biochemicals, Freehold, New Jersey), and digested for 20 min at 32°C, with continuous agitation. To disrupt rosettes, 0.2 ml of 0.99 M pH 7.2 EDTA (ethylenediamine tetraacetate) was then added and incubation with agitation continued for 5 min at 32°C. Undigested fragments were then removed by filtering through a fine stainless-steel mesh sieve, and the digest made up to 6 ml with RPMI 1640–1% FCS–0.01% DNAse.

To separate nurse cells from most mononuclear cells, unit-gravity zonal elutriation was immediately carried out on the EDTA-treated filtered digest, without prior centrifugation. The technique was as described previously (Andrews and Shortman, 1985; Shortman et al., 1989), but using the elutriation chamber of shape and dimensions shown in Fig. 2. The elutriation medium was stabilized by a shallow density gradient. The gradient-generation system and the gradient shape was as described in the references before, but the medium was more dense and more viscous. The elutriation medium consisted of 50% FCS, 50% mouse osmolarity buffered balanced salt solution (BSS) containing varying levels of metrizamide, so the final stabilizing density gradient was from 1.027 toward 1.040 g/cm$^3$ at 4°C; Metrizamide (Nyegaard, Oslo) was prepared as a stock solution in water (24.3% w/v; 1.125 g/cm$^3$) for addition to the balanced salt solution. The elutriation was run in a cold room at 4°C. The digest was placed directly in the lower conical half of the chamber, the upper half of the chamber screwed on until sealed, and the elutriation medium gradient pumped in at the bottom underneath the digest at 180 ml/hr. Fractions were collected as medium began to exit from the top of the chamber. When the nurse cells began to exit (at approximately 20 ml; Fig. 3), a higher pumping rate could be used and, because a continuing density increase was then no longer needed to stabilize the separation, the dense-medium reserve flask could be run dry, saving serum and Metrizamide. The fractions were screened directly under inverted phase microscopy, and the nurse-cell-enriched fractions (corresponding to elution volumes 25–120 ml in Fig. 3) were pooled.

To remove residual mononuclear cells, they were coated with a cocktail of mAb specific for T cells and myeloid cells, and then depleted using magnetic beads. The pooled nurse-cell-enriched elutriation fractions were underlaid with FCS-EDTA (90% FCS–10% 0.99M EDTA) to prevent any rosette reformation, and centrifuged to a small pellet. The cell pellet was resuspended in the mAb cocktail and incubated at 4°C for 25 min. The cocktail consisted of mAb specific for CD3 (KT3), CD4 (GK1.5), CD8 (53.6), Thy-1 (30H12), Mac-1 (M1/70), macrophage marker (F4/80), and

Gr-1 (RB68C5); they were used as supernatants of hybridomas grown in this laboratory, and were titrated prior to use. The cells were washed by suspension in BSS–10% FCS, underlaying with FCS–EDTA and centrifugation to a pellet. The pellet was resuspended in 200-$\mu$l BSS–FCS, and added to a pellet of washed antiimmunoglobulin-coated magnetic beads (Dynabeads M-450, Dynal, Oslo), at a ratio of four beads per cell, in a small (0.9-ml) siliconized-glass tube. The suspension was incubated for 20 min at 4°C with just sufficient rotational mixing to keep beads and cells in suspension, by sealing the small glass tube within a 5-ml plastic tube that was then rotated at an angle on a spiral mixer. The cells and beads were then resuspended in 4 ml BSS–FCS, and the beads removed using a magnet (MPC-1, Dynal); two bead removal cycles were used. The TNC were then recovered by centrifugation to a (very small) pellet in a 3-ml conical base siliconized-glass centrifuge tube.

To remove debris and any residual magnetic beads, buoyant density separation was used. The separation medium was mouse osmolarity pH 7.2 metrizamide, density 1.083 g/cm$^3$ at 4°C; this was obtained by diluting the previous stock metrizamide with BSS. The TNC pellet was suspended in 0.3 ml metrizamide in a 0.9-ml siliconized-glass tube, a further 0.3 ml of metrizamide layered underneath, and 0.2 ml FCS layered above. This small tube was sealed inside a larger plastic centrifuge tube, and spun for 8 min at 1500 g, 4°C, in a swing-out centrifuge. The upper 0.5 ml was collected, diluted in BSS–FCS, and centrifuged (in the 3-ml conical-base tubes) to recover the TNC as a very small pellet.

### Release of TNC-L

The purified TNC were suspended in 1 ml RPMI-1640 (mouse osmolarity, with additional HEPES buffer pH 7.2) containing 10% FCS and 5×10$^{-5}$ M 2-mercaptoethanol, and incubated at 37°C in a 10% $CO_2$-in-air incubator, normally for 7 hr. For experiments where the rate of release was monitored, the TNC were incubated in the flat-bottomed wells of a 96-well culture tray, and the nurse cells examined at various times under inverted phase-contrast microscopy for release of lymphocyte contents. To collect TNC-L, the suspension was mixed by several passages through a syringe needle, the cells centrifuged to a pellet, and any dead cells and debris removed by a density cut, performed as before but with metrizamide medium density 1.089 g/cm$^3$.

### Immunofluorescent Staining and Flow Cytometric Analysis

The mAb used were prepared, purified, and conjugated in this laboratory. Anti-CD8 was 53-6.7 (Ledbetter and Herzenberg, 1979), conjugated to fluorescein isothiocyanate (FITC); anti-CD4 was GK1.5 (Dialynas et al., 1983), conjugated to allophycocyanin (APC); anti-CD3 was KT3 (Tomonari, 1988), conjugated to biotin, and used with phycoerythrin (PE) avidin (Caltag Laboratories, San Francisco) as a second stage. All staining was in 3-ml siliconized-glass centrifuge tubes with a narrow conical base to ensure recovery of the small cell pellet; all centrifugations were at 400 g for 7 min at 4°C. For three-color stains, the cells were first incubated with the biotinylated mAb for 20 min at 4°C, and then washed by diluting with BSS–2% FCS layering over BSS–25% FCS and centrifuging. The cell pellet was finally incubated (20 min, 4°C) with the two remaining directly conjugated mAb together with PE-avidin. After washing, the cells were suspended in 1 ml BSS–FCS containing 10 $\mu$g/ml of propidium iodide (PI) (Calbiochem, La Jolla, CA), and passed through a fine nylon gauze filter. Flow cytometric analysis was carried out on a FACStar Plus (Becton Dickinson, Mountain View, California), using four fluorescence channels (including a separate channel for PI) with appropriate compensations. Dead cells were excluded on the basis of low low-angle light scatter and bright PI staining, and most nonlymphoid cells were excluded on the basis of high low-angle and side scatter. Files of 10,000 to 50,000 cells were acquired for analysis.

### Direct Fluoresceination of Cells

Thymocytes (3×10$^8$) were incubated with 300-$\mu$g fluorescein isothiocyanate (Sigma Chemical Company, Missouri), in 9 ml BSS–1% FCS at room temperature for 20 min, with mixing. The cells were then washed three times (layering over and centrifuging through FCS), before addition to thymus digests to monitor the carryover of exogenous thymocyte contaminants in TNC preparations. The level of these fluoresceinated cells at various stages of the purification was determined using a fluorescence microscope.

### Electron Microscopy

Cells were fixed by resuspension of the cell pellet in 2.5% glutaraldehyde in 0.1 M sodium cacodylate for at least 1 hr. They were postfixed in 2% osmium tetroxide for 1 hr, dehydrated through graded acetone, and infiltrated with Spurr resin (Probing and Structure, Thuringowa, Queensland, Australia). They were then sectioned on a Reichert Ultracut E (Reichert, Vienna), stained with uranyl acetate and lead citrate, and then viewed using a Philips CM 12 (Phillips, Eindhoven, The Netherlands).

### ACKNOWLEDGMENTS

We thank Rosemary van Driel for the electron microscopy, Ralph Rossi, Robyn Muir, and Frank Battye for assistance with flow cytometry. This work was supported by the National Health and Medical Research Council, Australia.

*(Received September 1, 1992)*

*(Accepted November 13, 1992)*

### REFERENCES

Andrews P., and Boyd R. (1985). The murine thymic nurse cell: An isolated thymic microenvironment. Eur. J. Immunol. **15:** 36–42.

Andrews P., Boyd R., and Shortman K. (1985). The limited immunocompetence of thymocytes within murine thymic nurse cells. Eur. J. Immunol. **15:** 1043–1048.

Andrews P., and Shortman K. (1985). Zonal unit-gravity elutriation: A new technique for separating large cells and multicellular complexes from cell suspensions. Cell Biophys. **7:** 251–266.

Boyd R.L., Oberhuber G., Hala K., and Wick G. (1984). Obese strain (OS) chickens with spontaneous autoimmune thyroiditis have a deficiency in thymic nurse cells. J. Immunol. **132:** 718–724.

Davidson N.J., and Boyd R.L. (1992). Delineation of chicken thymocytes by CD3/T-cell receptor complex, CD4 and CD8 antigen expression reveals phylogenetically conserved and novel thymocyte subsets. Intern. Immunol., in press.

Dialynas D., Quan Z., Wall K., Pierres A., Quintans J., Loken M., Pierres M., and Fitch F. (1983). Characterization of the murine T cell surface molecule, designated L3T4, identified by monoclonal antibody GK1.5: Similarity of L3T4 to the human Leu-3/T4 molecule. J. Immunol. **131:** 2445–2451.

Fink P.J., Weissman I.L., Kaplan H.S., and Kyewski B.A. (1984). The immunocompetence of murine stromal cell-associated thymocytes. J. Immunol. **132:** 2266–2272.

Hugo P., and Petrie H.T. (1992). Multiple routes for late intrathymic precursors to generate $CD4^+CD8^+$ thymocytes. Adv. in Cell. Biol. **5:** 37–53.

Kyewski B.A. (1986). Thymic nurse cells: Possible sites of T-cell selection. Immunol. Today **7:** 374–378.

Kyewski B.A., and Kaplan H.S. (1982). Lymphoepithelial interactions in the mouse thymus: Phenotypic and kinetic studies on thymic nurse cells. J. Immunol. **128:** 2287–2294.

Kyewski B.A., Momburg F., and Schirrmacher V. (1987). Phenotype of stromal cell-associated thymocytes *in situ* is compatible with selection of the T cell repertoire at an "immature" stage of thymic T cell differentiation. Eur. J. Immunol. **17:** 961–967.

Ledbetter J.A., and Herzenberg L.A. (1979). Xenogeneic monoclonal antibodies to mouse lymphoid differentiation antigens. Immunol. Rev. **47:** 63–90.

Penninger J., Hala K., and Wick G. (1990). Intrathymic nurse cell lymphocytes can induce a specific graft-versus-host reaction. J. Exp. Med. **172:** 521–529.

Shortman K., Vremec D., D'Amico A., Battye F., and Boyd R. (1989). Nature of the thymocytes associated with dendritic cells and macrophages in thymic rosettes. Cell. Immunol. **119:** 85–100.

Tomonari K. (1988). A rat antibody against a structure functionally related to the mouse T-cell receptor/T3 complex. Immunogenetics **78:** 455–458.

van Vliet E., Melis M., and van Ewijk W. (1984). Immunohistology of thymic nurse cells. Cell. Immunol. **87:** 101–109.

Wekerle H., Ketelsen U.-P., and Ernst M. (1980). Thymic nurse cells. Lymphoepithelial cell complexes in murine thymuses: Morphological and serological characterization. J. Exp. Med. **151:** 925–944.

Wekerle H., and Ketelsen U.P. (1980). Thymic nurse cells–Ia bearing epithelium involved in T-lymphocyte differentiation. Nature **283:** 402–404.

